# Can AI Be Useful in the Early Detection of Pancreatic Cancer in Patients with New-Onset Diabetes?

**DOI:** 10.3390/biomedicines13040836

**Published:** 2025-03-31

**Authors:** Maja Mejza, Anna Bajer, Sora Wanibuchi, Ewa Małecka-Wojciesko

**Affiliations:** 1 Department of Digestive Tract Diseases, Medical University of Lodz, 90-153 Lodz, Poland; maja.mejza@student.umed.lodz.pl (M.M.); anna.bajer1@student.umed.lodz.pl (A.B.); 2 Aichi Medical University Hospital, Nagakute 480-1195, Japan; wanibuchi.sora@gmail.com

**Keywords:** PDAC, NOD, pancreatic ductal adenocarcinoma, artificial intelligence, diabetes mellitus, deep learning, machine learning, neural networks, risk prediction

## Abstract

Pancreatic cancer is one of the most lethal neoplasms. Despite considerable research conducted in recent decades, not much has been achieved to improve its survival rate. That may stem from the lack of effective screening strategies in increased pancreatic cancer risk groups. One population that may be appropriate for screening is new-onset diabetes (NOD) patients. Such a conclusion stems from the fact that pancreatic cancer can cause diabetes several months before diagnosis. The most widely used screening tool for this population, the ENDPAC (Enriching New-Onset Diabetes for Pancreatic Cancer) model, has not achieved satisfactory results in validation trials. This provoked the first attempts at using artificial intelligence (AI) to create larger, multi-parameter models that could better identify the at-risk population, which would be suitable for screening. The results shown by the authors of these trials seem promising. Nonetheless, the number of publications is limited, and the downfalls of using AI are not well highlighted. This narrative review presents a summary of previous publications, recent advancements and feasible solutions for effective screening of patients with NOD for pancreatic cancer.

## 1. Introduction

### 1.1. Pancreatic Cancer and New-Onset Diabetes

Pancreatic ductal adenocarcinoma (PDAC), which accounts for approximately 90% of pancreatic cancers [1,2], is undoubtedly one of the most lethal neoplasms with a 5-year survival rate not exceeding 12.8% [3]. Unlike other types of cancer, the survival rates of PDAC have not improved significantly over the years. Data from the United States of America show that over ten years, from the 2000–2004 period to the 2010–2014 period, the survival rate only rose from 7.2% to 11.5% [4]. Reasons for these unsatisfactory statistics are, among others, the tumor’s structural and molecular variability [5] and late diagnosis, as well as limited treatment options. Most common symptoms preceding diagnosis, which include changes in bowel habits, epigastric, back and shoulder pain, lethargy, dysphagia, nausea and bloating [6,7], are non-specific and thus likely to be overlooked. Moreover, these are occurring intermittently, which may delay seeking help [8].

**Figure 1:**
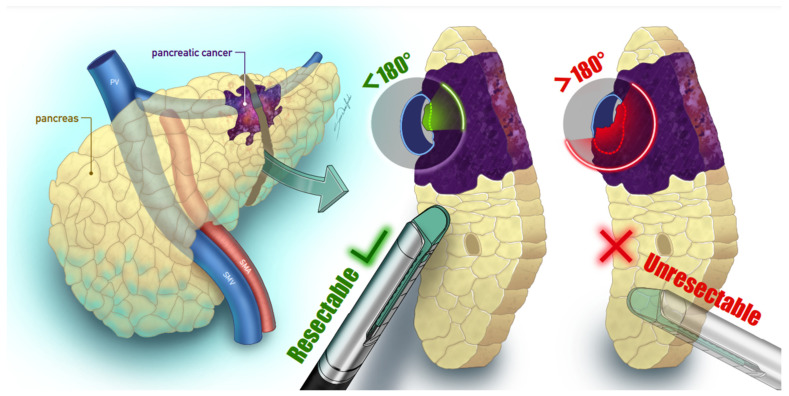
The resectability of pancreatic cancer is dependent on blood vessel encasement degree. Surgery is currently considered optimal when the invasion of surrounding major blood vessels is under 180 degrees [12], as it allows for avoiding the need for reconstruction.

Currently, only up to 20% of patients diagnosed with PDAC qualify for surgery [9], which is the only treatment offering a curative approach. According to the American Joint Committee on Cancer (AJCC), it can be performed in stages I–II. The only difference between stages Ia, Ib and IIa is the size of the tumor (T1, T2 and T3, accordingly). If the tumor extends beyond the pancreas without the presence of vascular involvement, it is classified as IIa. Stage IIb is characterized by node metastases with T1, 2 or 3, whereas in stage III the tumor invades the celiac axis or superior mesenteric artery (T4) [10]. Some cases of stages II and III can be considered borderline resectable [11], depending mostly on the level of vascular invasion. The non-operative approach is mostly adapted when there is over 180 degrees of encasement of the superior mesenteric artery, the superior mesenteric vein or the portal vein, all without the possibility of reconstruction [12] (Figure 1). The management of splenic vein (SV) invasion remains a subject of debate, as it is not currently classified as a resectability factor. Isolated SV invasion in early-stage cancer is uncommon [13]. Nonetheless, recent data show that it leads to a similarly poor prognosis as that of portal vein invasion [14]. Additionally, patients with SV encasement were shown to significantly benefit from pre-operative chemotherapy, which suggests the need for finding novel optimal resection guidelines for this group and highlights the importance of early diagnosis. The invasive stage may progress rapidly, further lowering the chances for successful treatment [15]. In patients who obtained successful resection, 5-year survival was reported to be 27%; meanwhile, the median survival of those at metastatic stages does not exceed six months [16].

Population-wide screening for pancreatic cancers is not recommended, since the disease is rare and proposed screening methods are highly invasive [17]. In fact, screening may lower the life expectancy of tested individuals due to false-positive results, followed by unnecessary surgeries [18]. Diseases which might be misdiagnosed as pancreatic cancer include, among others, chronic and autoimmune pancreatitis, cystic dystrophy in heterotopic pancreas, annular pancreas and accessory spleen, as well as adrenal and duodenal masses, which need careful diagnostics [19,20]. Thus, it is considered that surgical mortality outweighs the benefits of screening in an average-risk group [18].

According to the American College of Gastroenterology (ACG) and the American Gastroenterology Association (AGA), screening is strongly recommended only in patients with hereditary syndromes (Peutz–Jeghers syndrome, hereditary pancreatitis, familial atypical multiple mole melanoma) and individuals of familial pancreatic cancer kindreds whose first-degree relative was diagnosed with pancreatic cancer, as well as mutation carriers from BRCA1, BRCA2, PALB2, ATM and Lynch syndrome families, with a first- or second-degree relative diagnosed with the disease [21,22]. Data suggest that screening these patients might increase their life expectancy by up to 260 days [23]. Nonetheless, most cases of pancreatic cancers are sporadic [17]. Thus, new increased PDAC risk groups that could benefit from more precise observation are being explored. One of them is patients with diabetes mellitus (DM).

On the one hand, long-standing type 2 diabetes mellitus (T2DM) increases the risk of PDAC; on the other hand, diabetes mellitus may occur during PDAC [24]. However, multiple other pathologies of pancreatic diseases, such as chronic pancreatitis, cystic fibrosis and hemochromatosis, may also lead to DM [25]. In the case of PDAC, DM or impaired glucose tolerance is, at some point, present in 38.1–75% of patients [26,27,28,29,30]. Of particular interest in this setting is new-onset diabetes mellitus (NOD), which has been shown to be diagnosed up to 3 years before the cancer diagnosis [31]. NOD, in turn, is preceded by new-onset hyperglycemia (NOH) [32]. NOD constitutes up to 58% of diabetes cases in pancreatic cancer. In most cases, there are no cancer-specific symptoms at its onset. It was observed that the diagnosis of DM in patients with pancreatic cancer was often delayed or never made [33]. That implies the underestimation of NOD prevalence in pancreatic cancer. NOD was found to be successfully cured after pancreaticoduodenectomy in 57% of cases, further proving its origin [29]. Considering that some form of DM is present in almost half of patients with small, resectable tumors, pancreatic cancer-associated diabetes (PCD) seems like a perfect candidate for an early pancreatic cancer biomarker [34]. However, the risk of pancreatic cancer in NOD patients, though higher than in the general population, remains too low to implement surveillance in this group. According to Jensen et al. [35], among people with NOD who are 50 years or older, there is a 0.6% 3-year cumulative incidence of pancreatic cancer, which is too low for screening to be cost-effective. For cost-effectiveness of screening patients with NOD, the likelihood of PDAC being the cause of DM must reach at least 10% in the screening group [36]. Considering the aforementioned, there is still a growing need to find a better tool to predict the risk of PDAC in patients with NOD, and AI seems to be a promising candidate for that task. Narrowing the NOD population to the point where it is eligible for screening while still allowing early PDAC detection is still challenging and has been a subject of research.

The mechanism responsible for the connection of NOD and pancreatic cancer is yet to be fully explained. However, disruption of insulin and insulin-like growth factor (IGF) axes caused by primary chronic inflammation, fibrosis and sclerosis of parenchyma, as well as insulin resistance, can be identified as one of the potential crucial factors [37,38]. Physiologically, the synthesis of IGFs occurs mainly in the liver and serves as a stimulant for cellular proliferation [39]. IGF-1 and its receptor are known to be overexpressed in PDAC tissue, and their expression was correlated with cancer grade [40]. Additionally, tumor stroma is responsible for further secretion of IGF-1, worsening tumor aggressiveness. In fact, both IGF-1 and IGFBP-2 (insulin-like growth factor binding protein-2) were also suggested as possible biomarkers which could help to differentiate PDAC from healthy controls and patients with other diseases, such as chronic pancreatitis [41]. Insulin’s role in PDAC was also supported by the observation that drugs increasing insulin level (e.g., sulfonylureas and insulin analogues) are linked to increased risk of PDAC, whereas those reducing insulin resistance (e.g., metformin) have shown the opposite effect [37]. According to Wang et al. [42], another important factor may be the amyloid deposition present in the PDAC islet. It was suggested that the primary deposition leads to beta-cell dysfunction, resulting in DM. Additionally, substances, which have not yet been identified, secreted from pancreatic cancer cells could also be contributing to insulin resistance and impairing beta cells’ function [43].

The data search for this review was performed with the use of the keywords “artificial intelligence”, “machine learning”, “deep learning”, “pancreatic cancer” and “new-onset diabetes mellitus”, as well as their variations. The databases screened for publications referencing the given subject were PubMed, Scopus, Google Scholar and ClinicalTrials.gov. The literature included was published before February 2025. The research contains a summary of the current state of the art and future perspectives in pancreatic cancer screening in NOD patients, focusing on clinically feasible solutions.

### 1.2. AI in Cancer Diagnosis

Artificial intelligence (AI) is a broad term describing computer programs whose work can resemble one carried out by conscious or intelligent beings [44]. The part of AI responsible for allowing machines to learn and adapt to new experiences is regarded as machine learning (ML). Currently, in the medical field, the term AI is usually used to refer to ML-based algorithms, which are capable of learning with only minimal human intervention [45]. This may derive from the fact that many algorithms currently considered a part of the AI spectrum (e.g., logistic regression) were used in the medical field before the popularization of the name. ML has the reputation of being more flexible and interactive than classic statistical models. It can also be an excellent tool for better understanding casual relationships from non-randomized data sets [46]. ML algorithms usually start from a random guess. The accuracy is then asserted for later improvement [47]. Thus, applying an ML model begins with dividing data into training and validation sets.

**Figure 2:**
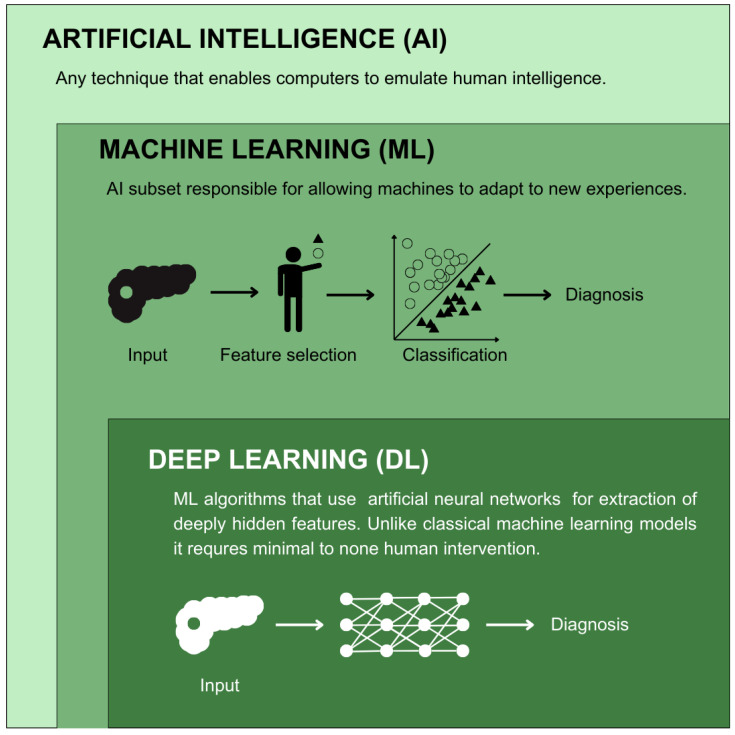
Key differences between terms commonly used to describe artificial intelligence.

Various divisions can be made to illustrate the AI algorithm landscape. The most basic of which is the difference between classical (shallow) ML, which needs human interventions for feature extraction, and deep learning (DL), which can work on unlabeled data sets with no or minimal need for human intervention [48] (Figure 2). In the case of shallow ML, the training set already contains the answer to the given problem question (e.g., in studies conducted retrospectively, the information on whether a given patient was diagnosed with pancreatic cancer in the observation period). By combining this answer with more data about a given case, the model can adapt to predicting outcomes in other patients. This ability is later assessed on a test data set to check how well the program predicts the outcomes with previously unknown cases. This is especially important in cancer risk assessment, where the models are prone to overfitting, meaning that the prediction is excellent upon training, up to a level that it cannot be generalized to other cases. This occurs because the model learns noise alongside the underlying patterns. To illustrate this, if an ML algorithm were to be trained on a data set where only men developed a cancer, it would likely fail to perform in a general population, because the information about sex could be overvalued or even critical for prediction. Overfitting is associated with small sample sizes, which are inadequate for the model to learn actual relations between the data [49]. Current applications of shallow ML in oncology include programs facilitating clinical decision making and prediction, with the most common techniques including gradient boosting (GB), random forests (RFs), support vector machines (SVMs), discriminant analysis, k-nearest neighbors (KNNs) and naïve Bayes (NB) [50,51,52].

As aforementioned, to estimate real cancer risk, one needs a certain amount of data about the patients. In technical nomenclature, these are often referred to as features, predictors or independent variables [53]. Thus, one of the processes most crucial for machine learning model elaboration is called feature engineering. It allows for the creation of new potential predictors from existing raw data. Newly created data representations are made to improve prediction accuracy. It may also serve simply as a means for better representation of the observed correlations. The detected features ought to be subsequently validated to assess their usefulness. However, feature engineering can only work well when the data set is diverse enough.

Unlike supervised methods, artificial neural networks (ANNs) do not require a human expert to perform feature engineering before model application because they can learn based on raw data [54] and automatically find new features. That makes DL a perfect choice in image analysis. The human eye often fails to fully capture all the data that could be extracted from an image [55]. Indeed, DL was previously shown to perform better in cancer diagnosis in histopathological [56,57] and radiological [58,59,60] image classification. Moreover, DL models are more flexible, which makes them easier to reuse for a broader range of purposes. That quality is notable in cancer screening, where an optimal model should be specific to more than one cancer type. This theory, however, does not always align with new analyses on the use of ML- and DL-based methods in cancer research. According to Painuli et al. [61], DL models currently dominate lung, liver, brain and skin cancer research, whereas ML algorithms are more often employed in studies regarding breast and pancreatic cancer.

## 2. Former Perspectives on Pancreatic Cancer Screening in Diabetic Patients

One of the most well-known tools for identifying patients with PCD is the Enriching New-Onset Diabetes for Pancreatic Cancer (ENDPAC) model. It includes information about recent deviations from earlier body weight and increases in glucose levels [62,63]. Sharma et al. [63] divided patients with NOD into three groups: an ENDPAC score ≥3 means a high risk of PDAC; a score of 1–2 means an intermediate risk; and a score ≤0 means a low risk. The model had a sensitivity of 78% and specificity of 85% in the initial validation cohort at a cut-off score ≥3. In the high-risk group, the 3-year incidence of PDAC was 3.6%, which is considerably more than 0.82% in the general NOD population. Patients in the low-risk group were assumed to have T2DM, due to high negative predictive value (NPV) [63]. One of the facts most crucial to the model is that before diabetes diagnosis, patients with PDAC tend to lose weight, whereas T2DM is associated with weight gain, which creates a strong, feasible discriminative factor. Moreover, the progression of their hyperglycemia is faster than in cases of T2DM [64].

ENDPAC’s predictive ability was lower in validation studies (AUROC: 0.72–0.75) [64,65], presumably due to larger cohorts. Establishing an accurate cut-off for implementing further testing is also an issue, as a cut-off >1 comes with insufficient specificity, while higher cut-offs decrease ENDPAC’s sensitivity. Nevertheless, the authors have proposed strategies which might resolve the problem, such as identifying patients with potentially false-positive results (e.g., recently using steroids with active malignancy or at the final stage of disease) [64].

Wang et al. evaluated the cost-effectiveness of risk-based PDAC screening using the ENDPAC model [66]. Among patients later diagnosed with PDAC, screening accounted for an additional 0.54 quality-adjusted life years (QALYs) due to an earlier diagnosis. In terms of finance, in the general NOD population, the best results showed USD 293 of additional expenditures with the risk-based screening strategy compared to no screening. This equaled a USD 65,076 per QALY gained, which is below the standard United States willingness-to-pay comparison threshold of USD 100,000 and thus can be deemed satisfactory [67]. The willingness-to-pay threshold is an approximate amount that a consumer of healthcare is ready to pay to gain a health benefit, considering other potential expenses burdening their resources [68]. The research was limited to United States healthcare; however, it managed to show the potential benefit in the establishment of a new screening method for the NOD population, especially with the new, potentially better-performing models.

Klatte et al. [69] instituted a model that combined the ENDPAC with family history screening. The authors created an application-based questionnaire including data about personal and family history of cancer aside from the information necessary for ENDPAC risk assessment, which was then filled by adult outpatients visiting the clinic of the Department of Gastroenterology and Hepatology of the Mayo Clinic Florida due to any reason between August 2018 and May 2019. As a result, about one-fourth (117/453) of patients screened were marked as having familial risk factors. Out of the identified 117 individuals, 49 completed genetic tests (5 of them underwent tests before the study), 9 of which were found to have a pathogenic gene variant of RET [c.1826G > A (p.Cys609Tyr)], ATM [c.7630 −2A > C (splice acceptor)], APC [c.3920 T > A (p.Ile1307Lys)] and SDH [c.91C > T (p.Arg31X)]. Out of these, variants of ATM and APC contribute to the development of PDAC. The patients who answered questions regarding ENDPAC NOD criteria 220/348 (63%) provided enough information to be eligible for NOD assessment. Out of this group, four patients met ENDPAC criteria, and two were later diagnosed with PDAC. This study has shown a way to easily narrow the screened group by identifying patients with a family history of cancer and new-onset diabetes, without significant financial contribution. However, such a method comes with limitations resulting from relying on a patient’s memory.

Before ENDPAC, Boursi et al. [70] developed another model for PDAC risk estimation in the NOD population within 3 years after diabetes diagnosis. The model was more complicated than ENDPAC, relying on age, BMI (Body Mass Index), change in BMI, smoking, use of proton pump inhibitors and anti-diabetic medications, HbA1c, cholesterol, hemoglobin, creatinine and alkaline phosphatase levels. The model’s AUROC was 0.82 (95% CI: 0.75–0.89). Sensitivity, specificity and PPV (positive predictive value) at the 1% risk cut-off were, respectively, 44.74%, 93. 95% and 2.6%.

Ali et al. [71] published another similar retrospective study on a nationwide Australian data set from 2002 to 2018. They searched for a better model to establish risk groups in pancreatic cancer. Their analysis was, however, restricted to Australian women with NOD and the follow-up was 3 years. Consistently with other studies, age ≥ 50 years and diabetes severity were the most important variables. The authors noticed a significant connection between treatment with insulin and pancreatic cancer. The change in anti-diabetic medication in such a short period indicates that fasting blood glucose was elevated despite treatment, which may be one of the factors suggesting pancreatic cancer as the cause of diabetes. The mean age at diabetes diagnosis was 75.2 for patients later diagnosed with pancreatic cancer, whereas for patients with T2DM it was 68.4. Interestingly, in this study pancreatic cancer was more often associated with the change in DM medication or insulin as the first medication (32% vs. 12%). This may be caused by the mechanisms responsible for diabetes in PDAC, which include not only insulin resistance but also the destruction of β-cells. In such cases, medication solely targeting insulin resistance might not be effective. The model’s discriminative ability defined with AUROC was 0.73 (95% CI 0.68–0.78). As much as the model seems less effective than the ENDPAC, it is unique in being based only on age and medication, which lowers the complexity, making it more accessible in clinical settings. The model’s positive predictive value (PPV) was two times higher than in the NOD population, which was considered still too low for implementing screening but combined with another biomarker has the potential to successfully narrow the screening population. Even though screening of patients older than 75 years old remains controversial [72], surgical resection of PDAC should not be excluded based solely on age. The 5-year survival rate of patients aged 70–79 who received this form of treatment reaches 15.6% and 11.3% in patients over 80. However, comorbidities and surgeon’s experience should be taken into consideration to make optimal decisions for every individual patient [73].

Another reliable, accurate PDAC biomarker could bring us closer to more cost-effective screening. Smith et al. [74] described complex study designs and strategies used for discovering such biomarkers. The heterogeneity of PDAC complicates finding a marker that could outperform carbohydrate antigen 19-9 (CA19-9) and be accepted by the FDA (Food and Drug Administration). Regrettably, despite having high sensitivity and specificity in symptomatic patients [75], CA19-9 lacks the PPV essential for screening purposes. Recent interest has been around an SRI gene encoding sorcin, a protein overexpressed in PDAC. Sorcin starts a pathway leading to the expression of inflammatory particles, such as plasminogen activator inhibitor-1 (PAI-1). PAI-1, identified in peripheral blood samples, was significantly elevated in individuals with PCD compared to T2DM patients. Thus, it can potentially be utilized as a biomarker [76]. However, further studies with larger cohorts are needed to validate those findings.

In the United States, 38% of the adult population is considered prediabetic [77], as evidenced with fasting plasma glucose levels of 100–125 mg/dL and/or HbA1c of 5.7–6.4% [78]. Elevated fasting blood glucose can be observed even 36 months prior to PDAC diagnosis [32]. Wu et al. [79] conducted a retrospective cohort study, where patients aged 50–84 with HbA1c ≥ 6.1% with no prior history of pancreatic cancer were divided into 12 cohort groups depending on HbA1c level and history of diabetes/elevated HbA1c. The 3-year PDAC incidence rate per 1000 patients reached 2.37 in non-Hispanic white patients with HbA1c of 6.5%, compared to 0.45 in the base cohort. Even though patients aged 50–84 with NOH have an elevated risk of pancreatic cancer, the risk is still not high enough to implement screening in this population based only on HbA1c level.

Therefore, multiple new prediction models have been developed to narrow down the population eligible for screening (Table 1). One of such models was presented by Boursi et al. [80], who conducted a retrospective cohort study with patients older than 35 years old with recently diagnosed fasting glucose levels of 100–125 mg/dL. The final model, consisting of information about age, BMI, use of proton pump inhibitors, levels of total cholesterol, low-density lipoprotein, alanine aminotransferase and alkaline phosphatase, achieved an AUROC of 0.71, 66.53% sensitivity, 54.91% specificity and 0.26 PPV at >0.1% cut-off, meaning that with this screening threshold, the model will detect even up to 66% of all PDAC cases diagnosed in 3 years after impaired fasting glucose was detected. This model includes more variables than ENDPAC, such as the use of proton pump inhibitors, which is associated with higher possibility of pancreatic cancer by causing hypergastrinemia [81]. In a meta-analysis, which included 10 studies, the use of PPIs (proton pump inhibitors) was linked to a 69.8% risk of pancreatic cancer. However, the number of studies and data quality do not provide certainty on that matter [82]. The increased value of another predictor, alanine aminotransferase (ALT), was linked to a moderately elevated risk of PDAC [83].

## 3. The Role of AI in the Identification of High-Risk Pancreatic Cancer Group

The first retrospective research, which aimed to implement AI algorithms to predict pancreatic cancer among those diagnosed with DM, was published in 2018 [84]. Hsieh et al. [84] collected data on patients diagnosed with T2DM between the years 2000 and 2012 from the Longitudinal Cohort of Diabetes Patients of the Taiwanese National Health Insurance Program. After excluding patients under 20 years of age and individuals with incomplete data, they gathered 1,358,634 participants. Only 3092 (0.23%) were diagnosed with pancreatic cancer in the follow-up period. This study has not set a definite endpoint but analyzed all available data, obtaining the mean follow-up period of 3.84 (SD = 3.44) years in the group diagnosed with pancreatic cancer and 6.87 (SD = 3.87) years in the second group. Key variables for the model’s implementation included demographic information, baseline comorbidities, Charlson comorbidity index, adapted Diabetes Complication Severity Index and medications. To evaluate the models, researchers have used sensitivity (recall), PPV (precision) and a harmonic mean of recall and precision (F1) (Table 2). Moreover, the AUROC was calculated between the actual outcome and the one predicted by the models, comparing classical linear regression and an artificial neural network (ANN), obtaining AUROCs of 0.707 (95% CI: 0.650–0.765) and 0.642 (95% CI: 0.576–0.708) accordingly. Even though both models achieved an identical precision of 99.5%, the sensitivity of the ANN model was lower (99.8% vs. 87.3%). This outperformance by linear regression may be explained by the fact that ANNs are usually more prone to statistical interferences. In the given case, the researchers have discussed the asymmetric outcome distribution as a main obstacle for the model. Another reason might be the logistic regression’s excellent suitability for handling categorical variables. This study has also provided an analysis of comorbidities, medications and other patients’ characteristics occurrence between the group with and without pancreatic cancer. Those with confirmed pancreatic cancer were characterized by a higher mean age (63.8 vs. 57.3) and more comorbidities (esp. acute and chronic pancreatitis, gallstones, cirrhosis, hyperlipidemia and obesity) but significantly fewer classic T2DM complications (retinopathy, nephropathy, peripheral vascular disease and neuropathy). The data were not only significant to the establishment of precise AI models but have also been a step towards a better understanding of PCD characteristics. The authors have nevertheless admitted that the relatively poor performance of the models might be related to the lack of data on lifestyle factors including alcohol consumption and smoking, which are known to influence pancreatic cancer risk [85].

A similar study was conducted more recently on retrospective records from 2009 to 2019 [86]. Data were gathered from the Taipei Medical University Clinical Research Database, which combines records from three major medical centers located in Taiwan (Taipei Medical University Hospital, Wan-Fang Hospital and Shuang-Ho Hospital). The final cohort consisted of 66,384 patients older than 40 and diagnosed with non-T1 DM. In total, 89 patients (0.13%) were subsequently diagnosed with pancreatic cancer within 4 years following an anti-diabetic medication prescription. The authors utilized eight different models (logistic regression, linear discriminant analysis (LDA), light gradient boosting machine (LGBM), gradient boosting machine (GBM), extreme gradient boosting (XGB), random forest (RF), SVM and ensemble voting (EV)) to compare their performance in risk prediction. The model that obtained the best results was LDA with a 0.9073 AUROC, 84% accuracy, 86% sensitivity and 84% specificity. The worst performance was of classical logistic regression (AUROC: 0.6669, 38% accuracy, 89% sensitivity and 38% specificity), which would support the notion that further exploration of novel machine learning techniques in the field may prove to be useful. The most important features of the winning model were the blood glucose level (HbA1c and glucose AC) within 12 months before the beginning of the study and hyperlipidemia. These results are significant and should be considered when designing further studies to optimize the results. The excellence of the obtained results may derive from technical factors, such as more advanced algorithms used, as well as from using data of higher dimensionality. This may explain why logistic regression was, unlike in the previous study, outperformed by novel models. Machine learning allows for efficient analysis of more complex patterns that would be too difficult to express in a plain statement.

Another analogous model was built by Cichosz et al. [87] using a random forest (RF) classification. The data set was based on a Danish nationwide cohort containing data from 1998 to 2018. The study was meant to predict the risk of PDAC in people with NOD to classify patients into low- and high-risk groups. The patients included were found by the ICD-10 (International Classification of Diseases Version 10) diagnosis code for DM or an ATC (Anatomical Therapeutic Chemical) code for DM medications. Patients with at least one code for T1DM or under the age of 50 at NOD diagnosis were subsequently excluded. NOD onset was defined as the first occurrence of one of the codes. Patients with the ICD-10 code for PDAC were identified from the obtained cohort. The authors have decided to further exclude all patients with a diagnosis of PDAC occurring before or up to 3 years after NOD diagnosis. This assumption is supported by research showing that NOD is associated with PDAC in this period [88,89], and the interval between NOD diagnosis and PDAC peaks at 32 months [90]. The establishment of the model was performed on 716 PCD and 716 T2DM individuals, and the data were restricted to sex, age and routine biochemical measurements as predictor variables. The tests analyzed were performed up to 3 years prior to their NOD diagnosis, and patients with missing data were excluded. Feature engineering was performed to show the underlying relations between the features and extract the most valuable measures. This was carried out by calculating the statistical features of the data (e.g., the mean and rate of change). Later, the importance of new features was calculated, extracting the best 20 features for model training. The final performance of the model on a test set was defined by AUROC 0.78 (95% CI: 0.75–0.83), which is comparable to previous models [80,91]. Similarly to other studies, older age and diabetes severity (rate of change in HbA1c) emerged as the most significant discriminators between T2DM- and PDAC-related diabetes. Other factors important for the analysis included altered trajectories of triglycerides, liver function and cholestatic parameter changes. These could be associated with the browning of adipose tissue and metabolism changes during PDAC progression [92]. Cichosz et al. [87] have moreover estimated the performance of a potential clinical-based surveillance program on 1 million patients with NOD. The model selected 1% of patients with the highest risk of PDAC. They have established that the relative risk (RR) of PDAC in that top 1% group is 20 times higher in comparison with the general population of patients with NOD. This is equal to a cumulative 3-year cancer risk of 12%, while in the general NOD cohort the risk is only 0.6%. Thus, such a program could serve as a means to reduce the number of patients that should undergo surveillance.

Clift et al. [93] decided to take a different approach and compared three models to assess their performance in assessing the individual risk of developing pancreatic cancer, regardless of histological type, within two years of DM diagnosis. The study was based on QResearch, a database gaining data from over 1500 general practices in the United Kingdom. Researchers have searched through the cases of patients registered between 2010 and 2021. Inclusion criteria involved 30–85 years of age and a new diagnosis of T2DM (based on SNOMED codes). Patients with a previous history of pancreatic cancer or prescriptions of anti-diabetic medications before the NOD diagnosis were excluded. The candidate predictors, identified from the literature, were subsequently evaluated with the use of factorial polynomials in Cox modeling. Those associated with a hazard ratio (HR) >1.1 or <0.9 with *p* < 0.01 were included in model creation. The presented ANN model had a Harrell’s C index of 0.650 (95% CI: 0.515–0.784), which was notably lower than both the Cox proportional hazards model (0.802) and ML algorithm XGB (0.723). Additionally, the statistical model explained 46% (95% CI: 43.1–48.9; PI: 39.3–52.7%) of the variation in time to pancreatic cancer diagnosis, meaning that the model could explain 46% of the differences between patients regarding when they were diagnosed. Moreover, the highest-risk 1% and 10% of predicted risks captured 12.51% and 44.06% of pancreatic cancer cases over 2 years. These results are especially significant when compared to a 3.95% sensitivity with the current recommendations in the United Kingdom, which advise an urgent abdominal imaging within two weeks in people over 60 with NOD and weight loss [93,94]. These results are crucial in displaying that not every problem requires a complex solution, as the traditional methods using a small set of clinical predictions can perform even better than novel XGB.

A comparison between the most well-known classic statistics-based models on the same data set was presented in the work of Khan et al. [95]. Their study aimed at designing a new XGB model to predict PDAC in retrospectively identified patients with NOD and compared the model to the older ones. The utilized data set was provided by TriNetX LCC, a global healthcare organizations network. Patients included in the study had the NOD diagnosis defined as an HbA1c level over 6.5%, with the first occurrence between 2009 and 2021. The initial cohort included 3,224,021 patients. Out of the three models, the one developed by the authors (XGB) has outperformed both END-PAC as well as the Boursi model, both considered promising in the field [96,97] (AUROC: 0.80 vs. 0.63 and 0.68, accordingly). To perform the analysis, researchers divided patients into three cohorts and measured all the variables necessary to perform previously described models. Consistent with previous data, patients with PCD were older, more anemic and weighed less than patients with T2DM. Additionally, they were found to have higher alkaline phosphatase levels than T2DM patients. Moreover, patients with T2DM were more often gaining weight before the onset of diabetes, whereas PCD patients often lost weight prior to the diagnosis. Anti-diabetic medications and proton pump inhibitors were more often prescribed to patients with PCD. History of malignancies and pancreatitis rates were also higher in the PCD group. These were used to analyze what parameters have influenced the model the most and can be used as a suggestion for further studies’ approach. This research has surely supported the arguments for the use of ML models in the field, underlining the importance of further assessment of their cost-effectiveness.

Applying RF, Chen et al. [98] presented another approach establishing three models that could work well in identifying PDAC among patients aged 50 to 84 without long-standing diabetes, who showed an elevated HbA1c level (≥6.5%). Further exclusion was based on previous history of pancreatic cancer or missing information crucial for meeting the inclusion/exclusion criteria. Data were gathered retrospectively from Kaiser Permanente Southern California, a community-based healthcare system. The analysis was conducted on entries between 2010 and 2018. An RF algorithm was applied to this study because authors described it as being helpful in avoiding the time-to-event-data analysis, characterizing more classical, regression-based models. The models’ ability to distinguish between patients that have and have not developed PDAC was characterized by an AUROC ranging from 0.808 to 0.822. The model with the highest AUROC used age, weight change and HbA1c change in the prior 18 months as predictors, with others only differing in the HbA1c change time frame. Generally, all three of the described models showed around 60% sensitivity and 80% specificity when targeting patients with the top 20% risk of developing PDAC. Generally, all three of the described models showed around 60% sensitivity and 80% specificity when targeting patients with the top 20% risk of developing PDAC. At this risk threshold, the model could identify over half of all the cases of PDAC. The focus put on the increase in HbA1c instead of increased blood glucose level may explain why the model performed better than the aforementioned END-PAC. Criteria based only on one laboratory test change (HbA1c) and demographic characteristics were less strict than the previously shown ML models, making the implementation easier in clinical settings. Implemented algorithms are stored on a website so that they can be reused and externally evaluated to fully explore their performance.

More recently, Sun et al. [99] pioneered combining clinical with genetic factors for the creation of five models (SVM, RF, XGB, logistic regression and multi-perceptron classifier (MLP)) assessing the risk of pancreatic cancer in patients with NOD. The cohort consisted of patients with T2DM or PCD, defined as having a pancreatic cancer diagnosis within 24 months after being diagnosed with DM. All data were gathered using UK Biobank and ICD-10 codes. Initially, 502,407 participants were enrolled, among which 25,897 were in the final NOD group. Only 100 patients were finally described as having PCD. To identify potential covariates for the model establishment, the researchers have performed an analysis of 82 candidate predictors including sex, age, weight, body size measurements, blood pressure, pulse rate, blood count and biochemical measurements, family history of smoking and drinking status, as well as systemic inflammatory markers, that they found to be connected to cancer risk in previous studies. Nevertheless, the model that obtained the best results integrated only the top five discriminatory measures (age, platelet count, systolic blood pressure, immature reticulocyte fraction and platelet crit) in combination with the genetic tests. Single nucleotide polymorphisms (SNPs), known for their association with pancreatic cancer, were investigated, two of which (rs6465133 (SRI) and rs61759623 (KRAS)) exhibited the potential to make a distinction between T2DM and PCD. An additional 22 promising SNPs were found in a further genome-wide association study analysis. The model incorporating both clinical and genetic factors obtained the best results, with the winning models being logistic regression (AUROC: 0.897) and the DL method, MLP (AUROC: 0.884). To increase the cost-effectiveness of such screening, the authors proposed a probability cut-off of 1.28%, which could, with the use of a winning model, lead to identifying 76% of PCD while testing only 13% of the NOD population. Limiting the screening group while maximizing the results was a strategy to ensure the model may be cost-effective in future evaluations. A higher, 5.26% risk threshold allows for the identification of 46% of PCD cases while testing only 2% of the NOD population over 3–6 months. This translates to a 98% specificity, 18.1% PPV, 99.6% NPV and 97.9% accuracy. The research is indicative of the potential of ML-driven models in recognizing the high-risk population. Nevertheless, external validation is necessary to fully understand its potential. The results of all these studies may differ significantly when the models are applied to a different population, as was presented previously. The utility of a screening model exists only when it is highly generalizable to all populations and does not only perform well on cases similar to the training data.

**Table 2:** Simplified comparison of the performance of different models in articles referencing the use of AI in identifying the high pancreatic cancer risk groups in the NOD population.

Study	AI		Non-AI	
	Models	Results	Models	Results
Hsieh et al. (2018) [84]	ANN	AUROC: 0.642 Precision: 0.995 Recall: 0.873 F1: 0.930	Logistic regression	AUROC: 0.707 Precision: 0.995 Recall: 0.998 F1: 0.996
Chen et al. (2023) [86]	SVM	AUROC: 0.7721 Precision: 0.0001 Recall: 0.7500 F1: 0.0003 Accuracy: 0.7409	Logistic regression	AUROC: 0.6669 Precision: 0.0001 Recall: 0.8889 F1: 0.0002 Accuracy: 0.3760
	LGBM	AUROC: 0.8632 Precision: 0.0002 Recall: 0.8333 F1: 0.0010 Accuracy: 0.7805		
	XGB	AUROC: 0.8772 Precision: 0.0002 Recall: 0.8611 F1: 0.0009 Accuracy: 0.8375		
	RF	AUROC: 0.8860 Precision: 0.0002 Recall: 0.8611 F1: 0.0015 Accuracy: 0.8336		
	GBM	AUROC: 0.9000 Precision: 0.0002 Recall: 0.8889 F1: 0.0008 Accuracy: 0.8102		
	Voting	AUROC: 0.9049 Precision: 0.0002 Recall: 0.8889 F1: 0.0009 Accuracy: 0.8373		
	LDA	AUROC: 0.9073 Precision: 0.0002 Recall: 0.8611 F1: 0.0012 Accuracy: 0.8403		
Cichosz et al. (2024) [87]	RF	AUROC: 0.78	-	-
Clift et al. (2024) [93]	ANN	Harrell’s C index: 0.650 Calibration slope: 1.855 CITL: 0.855	Cox proportional hazard modeling	Harrell’s’ C index: 0.802 Calibration slope: 0.980 CITL: −0.020
	XGB	Harrell’s C index: 0.723 Calibration slope: 1.180 CITL: 0.180		
Khan et al. (2023) [95]	XGB	AUROC: 0.800 Precision: 0.012 Recall: 0.750 Accuracy: 0.700	ENDPAC *	AUROC: 0.630 Precision: 0.008 Recall: 0.510 Accuracy: 0.700
			Boursi model *	AUROC: 0.680 Precision: 0.011 Recall: 0.540 Accuracy: 0.770
Chen et al. (2023) [98]	RF	AUROC: 0.808–0.822	-	-
Sun et al. (2024) [99]	RF	AUROC: 0.776	Logistic regression	AUROC: 0.897
	XGB	AUROC: 0.824		
	SVC	AUROC: 0.837		
	MLP	AUROC: 0.884		

Abbreviations: AI—artificial intelligence; ANNs—artificial neural networks; AUROC—area under the receiver operating characteristics curve; CITL—calibration-in-the-large; ENDPAC—Enriching New-Onset Diabetes for Pancreatic Cancer; GBM—gradient boosting machine; LDA—linear discriminant analysis; LGBM—light gradient boosting machine; MLP—multilayer perceptron; NOD—new-onset diabetes; RF—random forest; SVC—support vector classifier; SVM—support vector machine; XGB—extreme gradient boosting. *—The study has used models created by Sharma et al. (ENDPAC) [63] and Boursi et al. [70] instead of establishing novel models based on traditional statistical methods.

Bao et al. [100] tried a different approach and aimed at the differentiation of new-onset T2DM from PCD from a perspective of pancreatic hormones response. Recruited patients with the diagnosis of pancreatic cancer (with normal blood glucose or DM) underwent a ≥10 h fasting period before a mixed meal tolerance test (MMTT). It is an alternative to the currently recommended oral glucose tolerance test (OGTT) [101], where instead of glucose only, patients are ingesting complex foods containing fats, proteins and carbohydrates [102]. Another group of patients, healthy volunteers and those with NOD but without a previous history of pancreatic cancer, were recruited as controls. The algorithms applied were based on the results of insulin sensitivity and insulin secretion, as well as on a pancreatic polypeptide, a parameter correlated with the development of several pancreatic diseases including endocrine tumors [103]. Patients with PCD were presenting significantly weaker insulin and C-peptide responses to MMTT than controls. Contrarily, the PCD group had significantly better insulin sensitivity than the T2DM group, and a poor response to glucose stimulation was caused by lower insulin secretion. For a discriminative model to differentiate between the groups, researchers have compared four candidate models, RF, logistic regression, SVM and Naïve Bayes, the last of which had the highest obtained AUROC of 0.965, a classification accuracy of 81.5% and specificity of 92.2%. These results seem promising; nonetheless, the research was presented in the form of a meeting abstract; thus, the methodology cannot be fully assessed.

## 4. Practical Challenges in Implementing AI-Based Technologies

The clinical application of these novel AI technologies remains challenging for multiple reasons. Firstly, some of them, especially DL-based models, operate as “black boxes”, which means the user does not obtain outlook on the reasoning and calculations behind the models’ decisions [104]. That makes their usefulness hard to translate into clinical settings and provokes legal challenges. For instance, the European Union’s General Data Protection Regulation (GDPR) regulates some aspects of this issue by stating that every person has the right to receive information regarding the scope and purpose of processing data that concern them [105,106]. Moreover, the information should be conveyed “in a concise, transparent, intelligible and easily accessible form, using clear and plain language”, which is demanding or even impossible with the use of some of the aforementioned algorithms.

Another legal problem arises from the protection of data. The right to privacy is a fundamental right [105]. However, with the rapid progress in the use of new technologies and the need for large data sets [52], new threats to this right emerge before appropriate regulations. One such issue seems to be the responsibility for potential data leaks and errors [107]. AI can learn and make decisions on its own. To some extent, these decisions can be liable regardless of clinicians or developers. Surely, the limitation of having to consider the program as a “moral” or “responsible” entity can be avoided by integrating the techniques into clinicians’ practice rather than trying to replace human decision making [108]. In this way, the program could be treated only as a tool for physicians.

To address the legal challenges and ensure patients’ security, the FDA together with Health Canada and the United Kingdom’s Medicine and Healthcare Products Regulatory Agency (MHRA) have proposed 10 simple guiding principles on Good Machine Learning Practice (GMLP) for Medical Device Development Guiding Principles [109]. Nonetheless, there is still a lack of clear and precise policy on the approval of AI-based cancer screening programs. In August 2024, the European Union’s first-ever legal framework on AI development, the AI Act, entered into force [110]. However, its implication for the future of the healthcare industry remains unclear [111].

The scarcity of precise regulations may lead to a lower perception of these technologies by both patients and clinicians. Indeed, in previously published surveys, the main concerns expressed by oncologists were misleading diagnosis and treatment, overreliance on AI, algorithm or data bias, patient privacy and data security, the delay in the adaptation of laws, regulations and policies, the chance of damaging patients’ confidence in treatment with conflicting recommendations and analysis with clinicians, low flexibility to all situations and the potential difficulty posed to the patients with the need of adaptation to new circumstances [112,113]. The ethical dilemma of responsibility for negative outcomes resulting from decisions made with the use of AI [114] was further highlighted in 2021. Tamori et al. revealed that 73.5% of doctors expressed concern about that matter [115]. Even though clinicians show optimism towards AI, the majority of them do not believe that hospitals are ready to implement it in practice [116].

## 5. Gaps and Future Directions in PCD Screening

Out of the currently researched methods, explainable AI (XAI) seems to be an excellent answer to some of the previously described challenges. XAI is trying to establish models that will be easily understandable while keeping up their good performance [117]. To achieve this, researchers simplify the architecture and provide reasoning and understandable explanations behind the obtained results [118]. The simple post hoc clarification of feature relevance was already used in publications regarding the use of AI in PCD screening. In groups other than NOD, the research has previously implemented a specific technique, Shapley additive explanations (SHAPs), for displaying variables significance in PDAC risk prediction [119]. In PCD screening, this method, considered a gold standard, was implemented only by 3 authors [85,94,98]. Moreover, most of the papers were missing the explicit explanation behind the processes following feature choice. Another point that was often missed in the summarized studies yet is considered one of the four principles of XAI [120] is the clear indication of knowledge limits—the strict conditions under which the model is expected to function well [119].

Another breakthrough aspect of the study by Sun et al. [99] is the integration of SNPs into the model. The optimal performance was achieved with the integration of all considered SNPs supporting the notion that even though the effect of individual SNPs is small, combining them in polygenetic-risk scores could enhance the precision of future models [119]. The use of XAI in omics data was recently comprehensively described by Toussant et al. [117]. Among the suggested directions, an interesting point was made to highlight that explanations should be provided by text but also examples.

Federated learning (FL) is a different method that could further help with legal challenges, especially regarding data privacy assurance [121]. The idea behind it is to train the model without the exchange of raw data between institutions, as it is the most unsecure step in model establishment. This could be carried out by performing some steps locally at healthcare institutions that have gathered data. In that situation, only processed parameters are sent outside the institutions, minimizing the risk of personal information leaks. The coordinator that receives these paraments is then responsible for the global model creation [122]. The use of FL techniques could ensure the safety of patients, open up databases previously closed due to privacy concerns and facilitate the approval of medical agencies.

An alternative emerging direction is the incorporation of self-supervised learning (SSL) [123,124]. It was created for the model to learn from unlabeled data, diminishing the need for human data curation. Considering the amount of data that is passing through hospitals every day, this method is currently regarded as a very promising tool for healthcare. In the case of PCD screening, SSL could be especially useful due to the commonness of T2DM, which results in enormous unlabeled data sets for researching new discriminatory factors.

In recent years, implementations of AI have started to revolutionize image-based diagnosis. Nonetheless, these algorithms have not yet been integrated with biochemical model screening for PCD. With the ever-growing number of publications, including those concerning XAI, in medical image analysis [125], this is surely a subject that needs to be addressed in further research. AI tools have been previously shown to have excellent results in scanning for PDAC on Computed Tomography (CT) scans (AUROC: 0.97; sensitivity: 88%; specificity: 95%) [126] and endoscopic ultrasound (EUS) images (AUROC: 0.95; sensitivity: 93%; specificity: 90%) [127]. Moreover, trials of integrating DL for T2DM detection on CT scans have also been published [128,129], further promoting the need for combined clinical data, biomarkers and imaging integration in novel programs.

A further challenging aspect that should be addressed in future studies is the appropriate validation of emerging and existing technologies. The majority of the presented studies did not check the generalizability to other groups. Without proper external validation, results can be overfitted and cannot be extrapolated into other populations [130]. The most common technique, cross-validation, can generate results that are too optimistic, as a given set can have one feature that a model can learn to always perform well on the set population [131]. Cross-validation was present in the described studies. Nonetheless, Sun et al. once again presented an alternative with adjustments, nested [99], which is known to diminish such risks. Other methods for improving the classification validation are k-batch, ordered k-batch and balanced k-batch cross-validation [131].

Choosing an optimal high-risk group with AI is thought to help with cost-effectiveness; however, none of the models for PCD screening were assessed from this perspective. New trials analyzing how similar technologies perform in real-life circumstances are needed to fully understand the subject.

Lastly, referring to the AI extension of Consolidated Standards of Reporting Trials (CONSORT-AI) [132] and Transparent Reporting of a multivariable prediction model for Individual Prognosis or Diagnosis (TRIPOD) [133] guidelines is recommended for optimal reporting of future trials. Out of the described studies, only two [87,93] referred to one of them, the TRIPOD.

## 6. Conclusions

This review aimed to condense the existing knowledge on the usefulness of AI algorithms in screening for pancreatic cancer in patients with NOD. AI models perform comparably to non-AI models, suggesting these might be a reasonable alternative to consider. However, only two studies have presented results that outperform traditional statistical models.

Nevertheless, the limitations of this study cannot be omitted. Firstly, the number of articles matching the inclusion criteria is too small to draw far-reaching conclusions. Moreover, researchers vary greatly in both their results, as well as in their conclusions from obtained data. In addition, the AI model which presented the highest AUROC in differentiating PCD and T2DM was the one published only in the form of a conference abstract. Thus, not all data regarding its usability and performance were retrieved. It is also important to note that with the given data one cannot choose the optimal model for further research, as none of the aforementioned algorithms can be seen as generally superior to others. This was described by Wolpert et al. as the “No Free Lunch” (NFL) theorem [134]. It explains that in order to choose the best possible framework, one must previously understand the problem and data which are to be analyzed.

Unfortunately, novel AI-based models often involve a high level of complexity, which makes their results and data on which they operate harder to translate to real clinical usefulness [135]. This is especially true with the use of advanced feature engineering methods. The complex nature of these models is also provoking legal issues regarding the use of patients’ data and responsibility for potential errors. Novel techniques, such as XAI or FL, have the potential to change this by easing models’ interpretability and securing the information interchange.

The above should summarize existing knowledge and fields that are yet to be researched. AI models have presented a moderately optimistic perspective on the future of screening at-risk patients, showing results comparable to older statistic models. However, unlike the previous techniques, ML allows for finding new relations between the data, which humans are unlikely to perceive. Moreover, as the name suggests, it can learn from the data. This in turn makes these models adjustable to new clinical situations. Nevertheless, external validation on new, larger data sets, cost-effectiveness analyses and modeling the optimal risk triaging criteria are needed to fully assess their usefulness.

## Figures and Tables

**Table 1:** Summary of previous models established for PCD screening.

Study	Data Source	Objective	Population Characteristics	Data Needed	Performance
Sharma et al. (2018) [63]	The Rochester Epidemiology Project	Determining risk of pancreatic cancer in NOD patients	≥50 years old who met the glycemic criteria of NOD	Age at onset of diabetes, weight alterations from onset, change in blood glucose over 1 year before NOD	Sensitivity: 78% Specificity: 82% (initial validation cohort) AUROC 0.72–0.75 (validation studies [64,65])
Boursi et al. (2016) [70]	THIN database	Determining risk of pancreatic cancer in NOD patients	≥35 years old at the time of NOD diagnosis	Age, BMI, change in BMI, smoking, use of proton pump inhibitors and anti-diabetic medication, HbA1c, cholesterol, hemoglobin, creatinine and alkaline phosphatase levels	Sensitivity: 44.7% Specificity: 94% AUROC: 0.82
Ali et al. (2024) [71]	IMPROVE data set	Determining risk of pancreatic cancer in women with NOD	≥50-year-old women with diagnosed NOD	Age at NOD diagnosis, severity of diabetes, use of prescription medication	Sensitivity: 69% Specificity: 69% AUROC: 0.73
Boursi et al. (2022) [80]	THIN database	Determining risk of pancreatic cancer in patients with prediabetes	≥35 years old at the time of impaired fasting glucose diagnosis (100–125 mg/dL)	Age, BMI, use of proton pump inhibitors, total cholesterol, LDL (low-density lipoprotein), alkaline phosphatase, ALT (alanine aminotransferase)	Sensitivity: 66.53% Specificity: 54.91% AUROC: 0.71

## Abbreviations

The following abbreviations are used in this manuscript:

AI	Artificial intelligence
NOD	New-onset diabetes
PDAC	Pancreatic ductal adenocarcinoma
AJCC	American Joint Committee on Cancer
SV	Splenic vein
ACG	American College of Gastroenterology
AGA	American Gastroenterology Associations
DM	Diabetes mellitus
T2DM	Type 2 diabetes mellitus
NOH	New-onset hyperglycemia
PCD	Pancreatic cancer-associated diabetes
IGF	Insulin-like growth factor
IGFBP-2	Insulin-like growth factor binding protein-2
PAI-1	Plasminogen activator inhibitor-1
ML	Machine learning
DL	Deep learning
GB	Gradient boosting
SVM	Support vector machine
KNNs	K-nearest neighbors
NB	Naïve Bayes
ANNs	Artificial neural networks
ENDPAC	Enriching New-Onset Diabetes for Pancreatic Cancer
QALYs	Quality-adjusted life years
BMI	Body Mass Index
PPV	Positive predictive value
LDL	Low-density lipoprotein
CA19-9	Carbohydrate antigen 19-9
FDA	Food and Drug Administration
PPIs	Proton pump inhibitors
ALT	Alanine aminotransferase
LDA	Linear discriminant analysis
GBM	Gradient boosting machine
XGB	Extreme gradient boosting
LGBM	Light gradient boosting machine
RF	Random forest
RR	Relative risk
EV	Ensemble voting
ICD-10	International Classification of Diseases Version 10
ATC	Anatomical Therapeutic Chemical
MPL	Multi-perceptron classifier
SNP	Single nucleotide polymorphism
NPV	Negative predictive value
MMTT	Mixed meal tolerance test
OGTT	Oral glucose tolerance test
GDPR	General Data Protection Regulation
MHRA	Medicine and Healthcare Products Regulatory Agency
GMLP	Good Machine Learning Practice
XAI	Explainable AI
FL	Federated learning
SSL	Self-supervised learning
CT	Computed Tomography
EUS	Endoscopic ultrasound
CONSORT-AI	Consolidated Standards of Reporting Trials AI Extension
TRIPOD	Transparent Reporting of a multivariable prediction model for Individual Prognosis or Diagnosis
NFL	“No Free Lunch” (theorem)
